# Nicotinamide Enhances Repair of Arsenic and Ultraviolet Radiation-Induced DNA Damage in HaCaT Keratinocytes and Ex Vivo Human Skin

**DOI:** 10.1371/journal.pone.0117491

**Published:** 2015-02-06

**Authors:** Benjamin C. Thompson, Gary M. Halliday, Diona L. Damian

**Affiliations:** 1 Department of Dermatology, Sydney Cancer Centre, Bosch Institute, University of Sydney at Royal Prince Alfred Hospital, Camperdown, Sydney; 2 Melanoma Institute Australia, North Sydney, Australia; The University of Queensland, AUSTRALIA

## Abstract

Arsenic-induced skin cancer is a significant global health burden. In areas with arsenic contamination of water sources, such as China, Pakistan, Myanmar, Cambodia and especially Bangladesh and West Bengal, large populations are at risk of arsenic-induced skin cancer. Arsenic acts as a co-carcinogen with ultraviolet (UV) radiation and affects DNA damage and repair. Nicotinamide (vitamin B3) reduces premalignant keratoses in sun-damaged skin, likely by prevention of UV-induced cellular energy depletion and enhancement of DNA repair. We investigated whether nicotinamide modifies DNA repair following exposure to UV radiation and sodium arsenite. HaCaT keratinocytes and ex vivo human skin were exposed to 2μM sodium arsenite and low dose (2J/cm^2^) solar-simulated UV, with and without nicotinamide supplementation. DNA photolesions in the form of 8-oxo-7,8-dihydro-2′-deoxyguanosine and cyclobutane pyrimidine dimers were detected by immunofluorescence. Arsenic exposure significantly increased levels of 8-oxo-7,8-dihydro-2′-deoxyguanosine in irradiated cells. Nicotinamide reduced both types of photolesions in HaCaT keratinocytes and in ex vivo human skin, likely by enhancing DNA repair. These results demonstrate a reduction of two different photolesions over time in two different models in UV and arsenic exposed cells. Nicotinamide is a nontoxic, inexpensive agent with potential for chemoprevention of arsenic induced skin cancer.

## Introduction

Ultraviolet (UV) radiation is the primary carcinogen associated with skin cancer. Arsenic, in contaminated groundwater used for drinking and crop irrigation, is also a significant cause of skin cancer globally. More than 150 million people in Bangladesh and West Bengal are at risk of arsenic-induced skin cancer. In addition, 21% of people from arsenic affected districts of Bangladesh were found to have dermatological signs of chronic arsenic toxicity [1]. Arsenic is also present at high levels in drinking water in Cambodia and Vietnam [2], China [3] and Taiwan [4]. The most common skin cancers induced by arsenic exposure are Bowen’s disease (squamous cell carcinoma in-situ), basal cell carcinoma and invasive squamous cell carcinoma [5]. Skin complications from arsenic exposure develop after a 5–10 year latency period [6] and continue to develop decades after cessation of arsenic exposure [7], suggesting that long term prevention would be beneficial.

Ultraviolet radiation induces DNA damage in keratinocytes directly in the form of cyclobutane pyrimidine dimers (CPDs), and through production of reactive oxygen species (ROS) with the subsequent formation of oxidative DNA damage such as 8-oxo-7,8-dihydro-2’-deoxyguanosine (8oxoG). Failure to repair these DNA lesions can result in genetic mutations [8].

Arsenic is thought to damage DNA and to impair DNA repair. In murine models, arsenic acts as a co-carcinogen in the skin, with a 2.4-fold increase in skin cancers seen in mice exposed to both UV and 10mg/L sodium arsenite in drinking water for 26 weeks, compared to UV irradiation alone. No skin cancers developed in unirradiated, arsenic-exposed mice [9]. Furthermore, in humans, arsenic-induced Bowen’s disease contained higher levels of 8oxoG compared to non arsenic-induced Bowen’s disease [10].

HaCaT keratinocytes incubated with 10–30μM sodium arsenite have significantly greater levels of 8oxoG than controls [11], although this concentration of arsenic also affects cell viability [12]. At clinically relevant, non-cytotoxic levels (<5μM), sodium arsenite alone did not increase 8oxoG. However, when HaCaT keratinocytes are exposed to both 2μM sodium arsenite and 8J/cm^2^ of UV, 8oxoG is significantly increased compared to UV alone [13]. Exposure of HaCaT keratinocytes to sodium arsenite also resulted in higher CPD levels 24 hours after UV radiation. This was thought to be related to inducible nitric oxide synthase, suggesting that this plays a role in CPD repair inhibition by arsenic [14].

Nicotinamide, an amide form of vitamin B3, is a safe, widely available and inexpensive agent that shows promise for skin cancer chemoprevention. In phase 2 clinical trials, oral nicotinamide reduced premalignant actinic keratoses in sun-damaged Australians over a 4 month period [15], and accelerated regression of keratoses when used topically [16]. UV-induced immunosuppression and DNA damage are key pathways in photocarcinogenesis. Nicotinamide prevents UV-induced immunsuppression in humans [17], increases unscheduled DNA synthesis (UDS; DNA repair) in HaCaT cells and enhances DNA repair in ex vivo human skin following UV irradiation [18]. Nicotinamide’s photoprotective effects are thought to reflect its ability to prevent UV-induced depletion of ATP [19], which compromises the energy-intensive process of DNA repair [20].

Here we show that nicotinamide reduces arsenic-induced DNA damage in irradiated HaCaT cells and in ex vivo human skin. Hence nicotinamide may prove a useful and clinically feasible agent for chemoprevention of arsenic-induced skin malignancy.

## Materials and Methods

### HaCaT cell line

HaCaT cells were maintained in Dulbecco’s Modified Eagle’s Medium (DMEM) supplemented with 10% fetal bovine serum (FBS) (Noble Park North, VIC, Australia), in a 37°C humidified incubator (5% CO_2_ in-air).

### Preparation of human skin for assays

Skin was obtained in accordance with the principles of the Declaration of Helsinki, with approval by the University of Sydney Human Ethics Committee from healthy volunteers undergoing elective plastic surgery. All volunteers provided written informed consent. Skin was trimmed and cut into 5x5mm samples and transferred to 60mm petri dishes containing DMEM supplemented with 10% FBS and penicillin-streptomycin-amphotericin B (10,000U/ml, 10,000μg/ml and 25μg/ml respectively (Gibco, Life Technologies, Calsbad, CA, USA). Skin samples were treated and fixed within 72 hours of surgical collection.

### Exposure to nicotinamide and sodium arsenite

**Fig 1:**
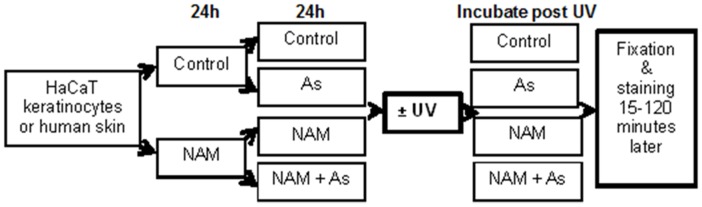
HaCaT human keratinocytes or ex vivo human skin samples were incubated ± 50μM nicotinamide (NAM) for 24h, and then incubated ± NAM and ± 2μM sodium arsenite (As) for a further 24h. Samples were then irradiated with a single, fixed dose of solar-simulated UV (2J/cm^2^), before once again being incubated ±NAM and ±As as per their pre-irradiation treatment allocation. The HaCaT cells were allowed to incubate for various periods before fixation; 15, 30, 45 and 75 minutes for oxidative DNA damage and 15, 45, 75 and 120 minutes for cyclobutane pyrimidine dimers. Skin samples were incubated for 45 minutes post-irradiation, when peak levels of DNA photolesions were observed.

A 5mM nicotinamide (Sigma-Aldrich, St Louis, MO, USA) in double distilled water solution and a PBS (phosphate buffered solution) control were blinded and diluted 1 in 100 with cell media to a final concentration of 50μM. The cells and skin were incubated for 24h (±nicotinamide) prior to sodium arsenite exposure. A sodium arsenite (Ajax, Thermo Fisher Scientific Inc., Waltham, MA, USA) diluted in double distilled water solution and a PBS control were blinded and diluted in media to a final concentration of 2μM. 4 blinded culture environments resulted: nicotinamide + arsenic, nicotinamide alone, arsenic alone, control. The cells and tissue were then incubated in these media for another 24h prior to irradiation (or kept unirradiated) and replaced with the same culture environments post irradiation (Fig. 1).

### UV Irradiation

Cells or *ex-vivo* human skin explants were irradiated with a 1000W xenon arc solar simulator (Oriel, Newport, Stratford, CT, USA). The UV spectrum and intensity was measured with a scanning spectroradiometer (OL 756, Optronic Laboratories Inc., Orlando, FL, USA). The spectrum is similar to sunlight [21] and the dose of 2J/cm^2^ is equivalent to 5–10 minutes of Sydney autumn sunlight. The irradiance was determined prior to each experiment with an IL–1700 broadband radiometer (International Light, Newburyport, MA) calibrated against the spectroradiometer.

### Viability of HaCaT cells following UV radiation and arsenite

HaCaT keratinocytes (250 000 cells) were plated into 60mm petri dishes. 24h later, the media was replaced with media containing 50μM nicotinamide or PBS. After a further 24h, the media was replaced with media containing 2μM sodium arsenite or PBS and 50μM nicotinamide or PBS. Cells were then exposed to 2J/cm^2^ of UV in 1ml of PBS. The PBS was then replaced by media containing the respective nicotinamide/arsenite/PBS and incubated for another 24h before determination of cell viability using a Vi-CELL cell counter (Beckman Coulter, Fullerton, CA, USA) based on an automated trypan blue staining procedure.

### Immunofluorescent detection of DNA damage (HaCaTs)

50,000 HaCaT cells/ml were seeded into 8 well chamber slides. 24h after seeding, the media was replaced with media containing 50μM nicotinamide. After a further 24h, the media was replaced with media containing both 50μM nicotinamide and 2μM sodium arsenite. After a further 24h incubation, cells were irradiated with 2J/cm^2^ of UV in PBS, or kept unirradiated. Cells were incubated post-irradiation for various times before fixation.

Immunofluorescent staining of 8oxoG and CPDs was performed as described previously [22] with modifications. After the specified time post irradiation, cells were fixed with 50:50 methanol and acetone for 10 minutes at -20°C. Slides were incubated with RNase A (Amresco, Solon, OH, USA) 100μg/L for 1h. This step was only included when staining for 8oxoG. 2N HCl was added to each well for 15 minutes and neutralized with 50mM tris base. Slides were then incubated for 30 minutes at 37°C with protein block (Dakocytomation, Glostrup, Denmark). For detection of 8oxoG, they were incubated with anti-8oxoG mouse monoclonal primary antibody (Trevigen, MD, USA) diluted 1:333 with antibody diluent (Dako, Glostrup, Denmark), for 1 hour. Isotype control replaced the primary antibody in control wells [mouse IgG2b isotype (Dakocytomation, Glostrup, Denmark)]. The secondary antibody alexa-fluor 594 (Invitrogen, Calsbad, CA, USA) diluted 1:200 with antibody diluent was used to visualize the staining.

For detection of CPDs, anti-thymine dimer antibody (Kamiya Biomedical Company, Seattle, WA, USA) was used for the primary antibody and IRDye 680LT (polyclonal) anti-mouse IgG (Licor, Lincoln, NA, USA) as the secondary antibody. Mounting media containing 4′,6-diamidino-2-phenylindole (DAPI) (1.5μg/ml) (Vector laboratories, Burlingame, CA, USA) was used for counterstaining. Images were analysed using Image-Pro Plus v7.0 software (Media Cybernetics, Bethesda, MD, USA). Three images were analysed per well, and the mean used for each of triplicate experiments. The DAPI image channel was used to identify cell nuclei. Subsequently the mean nuclear intensity per cell of the 8oxoG/CPDs was determined.

### Immunofluorescent detection of DNA damage in ex vivo human skin

Ex vivo human skin was incubated in media supplemented with 50μM nicotinamide (or PBS) for 24h. This was followed by incubation in 2μM sodium arsenite with additional 50μM nicotinamide (or PBS) for a further 24h. Skin was then washed with PBS and irradiated with 2J/cm^2^ of UV before further incubation in the same environments as pre irradiation. The samples were incubated for 45 minutes before fixation. The 45 minute time period was chosen as this allowed the keratinocytes to undergo partial but not complete DNA repair, so that a difference in DNA repair in the various groups was demonstrated by a difference in the levels of DNA damage over the timecourse.

45 minutes post-irradiation, the skin was fixed in 4% paraformaldehyde and embedded in paraffin. The paraffin embedded tissue was cut into 5μm sections onto Superfrost Plus microscope slides (Gerhard Menzel GmbH, Braunschweig, Germany). For detection of 8oxoG, slides were incubated with Proteinase K 40μg/ml (GE Healthcare, Buckinghamshire, U.K) for 30 minutes. For detection of CPDs, slides were heated for 12 minutes in 10mM sodium citrate solution (Ajax, Thermo Fisher Scientific Inc., Waltham, MA, USA) before cooling for 20 minutes to room temperature.

The slides were then stained in a similar manner to the HaCaT cells however for both primary antibodies, the IRDye 680LT secondary antibody was applied. All slides were blinded until image analysis was complete. A 20x objective was used and images were acquired. Folds, tissue end segments and unstained areas were avoided during image capture.

### Statistical analysis

Statistical analysis was performed using Prism 6.0 software (Graphpad Software Inc., La Jolla, CA USA). Unless otherwise specified, results are expressed as mean±SEM. For time courses, analysis was determined by repeated measured analysis of variance (ANOVA). For single time points, one way ANOVA with Bonferroni post-hoc test was used. In all analyses, p<0.05 was considered significant. Each experiment was repeated 3 times at each timepoint with all timepoints included in each experiment. The mean of the cells was regarded as the level of photodamage for that time point and results are shown as the mean of triplicate experiments.

## Results

### Cell viability is not affected by 2J/cm^2^ of UV or incubation with 50μM nicotinamide and 2μM sodium arsenite

**Fig 2:**
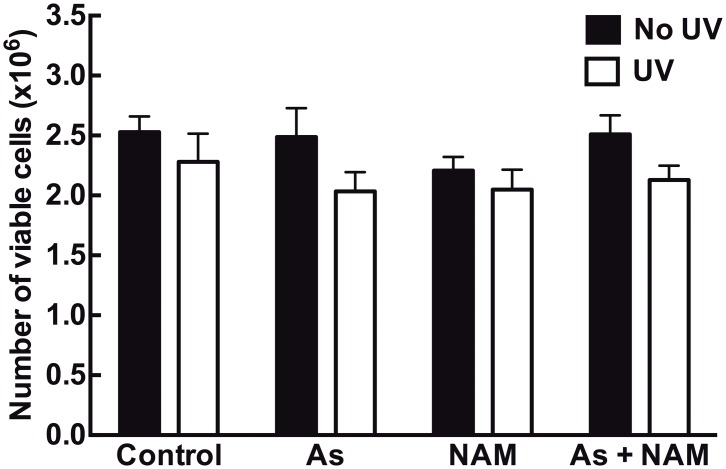
Viability of HaCaT keratinocytes is not affected by exposure to nicotinamide (NAM), sodium arsenite (As) and UV. HaCaT keratinocytes were exposed to 50μM nicotinamide, 2μM sodium arsenite or PBS (control) alone, with and without irradiation with 2 J/cm^2^ of UV. Columns represent the mean of the experiments ±SEM (n = 6). There were no significant differences detected between the groups p>0.05, one way ANOVA.

Viability of HaCaT keratinocytes was determined following exposure to nicotinamide, sodium arsenite and UV. There was no significant difference between the groups (n = 6, one way ANOVA) indicating that the interventions did not affect cell viability (Fig. 2).

### Nicotinamide reduces oxidative DNA damage in HaCaT keratinocytes following UV and sodium arsenite exposure

**Fig 3:**
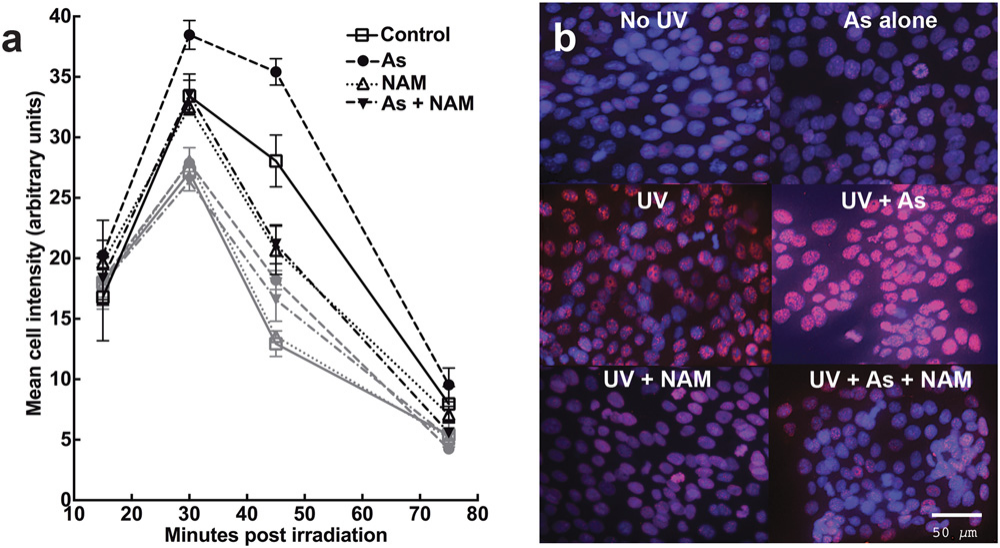
Nicotinamide (NAM) reduced arsenic- (As) and UV-induced 8oxoG in HaCaT keratinocytes (a). HaCaT keratinocytes were exposed to either 50μM nicotinamide, 2μM sodium arsenite, both sodium arsenite and nicotinamide or PBS (control) alone, with or without UV. Grey lines represent unirradiated groups. The mean of each timepoint is displayed ± SEM (n = 3). Significant differences included UV versus (vs) no UV (p<0.05), UV vs UV + As (p<0.01), UV vs UV + NAM (p<0.05) and UV + As vs UV + As + NAM (p<0.05) two way ANOVA. **Staining of 8oxoG 45 minutes following UV radiation and arsenic exposure in HaCaT keratinocytes (and in unirradiated keratinocytes) shows reduced 8oxoG in irradiated, nicotinamide treated cells (b)**. The blue staining represents DAPI (nuclear staining). The red staining represents 8oxoG staining.

UV exposure significantly increased 8oxoG levels over the timecourse studied (p<0.05; n = 3, repeated measures ANOVA) compared to the unirradiated group. Levels of 8oxoG initially increased as these photolesions were formed/detected and then decreased as they were repaired. Photolesion levels were significantly increased further by exposure to sodium arsenite compared to UV alone (p<0.01; n = 3; repeated measures ANOVA). 50μM nicotinamide significantly reduced the amount of 8oxoG over the timecourse compared to the UV exposed (p<0.05; n = 3) and UV plus arsenic groups (p<0.05; n = 3) (Fig. 3).

### Nicotinamide reduces CPDs in HaCaT keratinocytes following UV and sodium arsenite exposure

**Fig 4:**
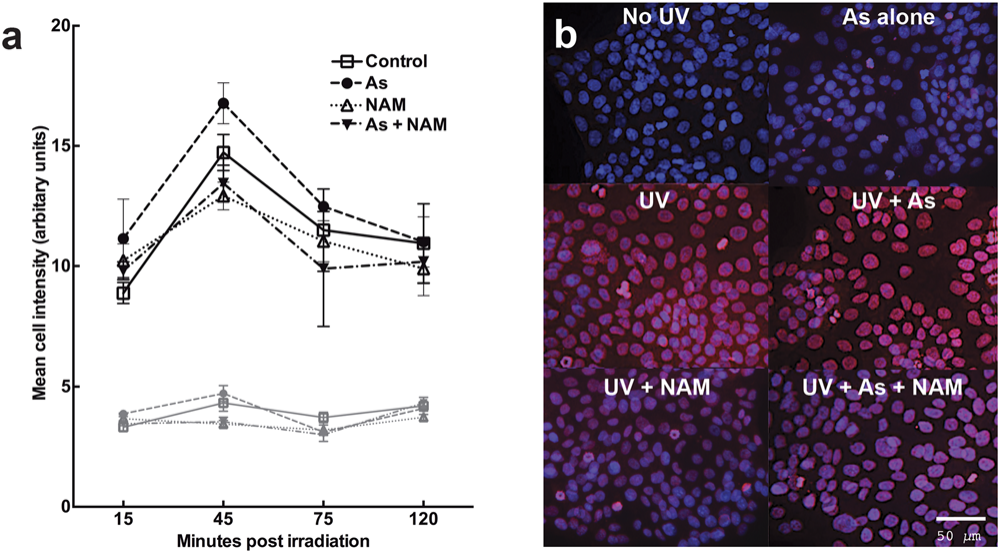
Nicotinamide (NAM) reduced arsenic (As) and UV-induced CPDs in HaCaT keratinocytes over time (a). The cells were stained for CPDs with grey lines representing unirradiated groups. The mean of each timepoint is displayed ± SEM (n = 3). Significant differences included UV vs no UV (p<0.01), UV vs UV + NAM (p<0.05) and UV + As vs UV + As + NAM (p<0.05) two way ANOVA. **Staining of CPDs 45 minutes following UV radiation, arsenic and nicotinamide exposure in HaCaT keratinocytes and in unirradiated keratinocytes (b)**. The blue staining represents DAPI (nuclear staining). The red staining represents CPD staining.

The UV exposed groups, as expected, had significantly greater staining than unirradiated groups (p<0.01; n = 3; repeated measures ANOVA). In all irradiated groups CPDs increased as they formed in the DNA and then decreased after as they were repaired. Exposure of irradiated cells to sodium arsenite did not increase CPDs further (p>0.05; n = 3). The addition of nicotinamide significantly reduced CPD levels in both the UV exposed (p<0.05; n = 3) and UV plus sodium arsenite groups (p<0.05; n = 3) (Fig. 4).

### Nicotinamide reduces DNA damage by UV irradiation and arsenic in ex vivo human epidermis

**Fig 5:**
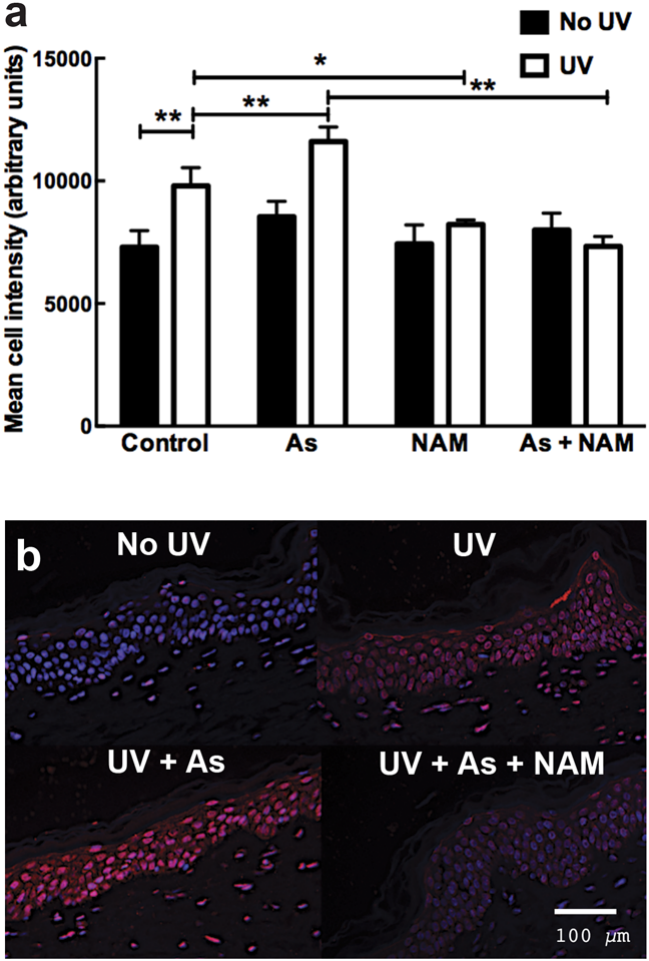
Nicotinamide reduced arsenic and UV-induced 8oxoG levels in the epidermal layer of *ex vivo* human skin at 45 minutes after UV irradiation (a). Samples were exposed to either 50μM nicotinamide, 2μM sodium arsenite, PBS (control) with or without 2J/cm^2^ of UV. The columns represent the mean ±SEM (n = 3). * = p<0.05, ** = p<0.01 one way ANOVA. **Staining of 8oxoG 45 minutes following UV radiation, arsenic and nicotinamide exposure in *ex-vivo* human skin (b)**. The blue staining represents DAPI (nuclear staining). The red staining represents 8oxoG staining.

UV irradiation significantly increased the amount of epidermal 8oxoG present compared to the unirradiated control (p<0.01; n = 3; one way ANOVA). This was further increased in the UV plus sodium arsenite treated skin compared to UV alone (p<0.01; n = 3). Both the nicotinamide plus UV (p<0.05; n = 3) and nicotinamide plus arsenic plus UV (p<0.01; n = 3) groups had significantly lower levels of 8oxoG at 45 minutes compared to the UV or UV plus arsenic groups (Fig. 5).

**Fig 6:**
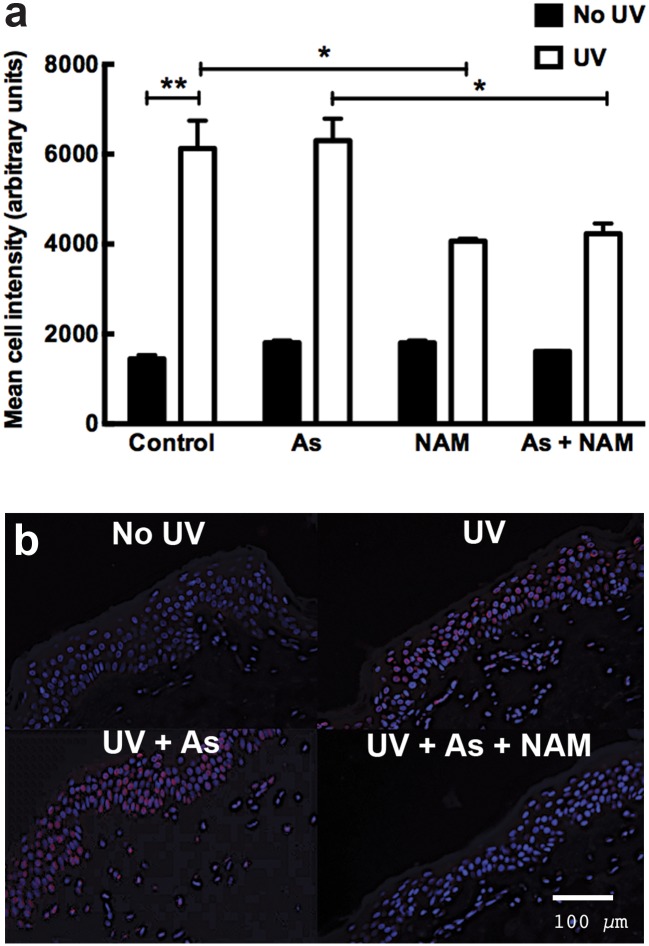
Nicotinamide reduced UV-induced CPDs in arsenic-treated and control irradiated *ex vivo* human skin at 45 minutes after UV irradiation (a). Samples were exposed to either 50μM nicotinamide, 2μM sodium arsenite, PBS (control) with or without 2J/cm^2^ of UV. The columns represent the mean ±SEM (n = 3). * = p<0.05, ** = p<0.01 one way ANOVA. **Staining of CPDs 45 minutes following UV radiation and arsenic exposure in *ex-vivo* human skin (b)**. The blue staining represents DAPI (nuclear staining). The red staining represents CPD staining.

Staining was also performed for CPDs at 45 min after exposure to allow time for repair to have commenced. Once again, irradiated skin had a greater level of epidermal CPDs compared to the unirradiated control (p<0.01; n = 3; one way ANOVA). This was not further increased by sodium arsenite. The nicotinamide plus UV (p<0.05; n = 3) and nicotinamide plus arsenic plus UV (p<0.05; n = 3) groups had significantly reduced levels of CPDs compared to the UV or UV plus arsenic groups (Fig. 6).

## Discussion

We found reductions in both 8oxoG and CPDs when arsenic and UV-exposed HaCaT keratinocytes were treated with nicotinamide. Furthermore, an increase in 8oxoG in irradiated, arsenic-exposed HaCaTs compared to UV alone was observed. The effect was measured over a timecourse, with no observed difference in photolesion levels at the first (15 minute) timepoint, suggesting that nicotinamide enhanced repair of damaged DNA rather than reducing photolesion production. This was further supported by studies in ex-vivo human skin.

The low, suberythemal UV dose used in these studies approximates 10 minutes of midday Autumn Sydney sunlight, mimicking incidental sunlight exposure [17]. The dose and form of arsenic used (2μM sodium arsenite) can be compared to water-source concentrations of arsenic that do not cause acute toxicity but lead to long term risk of cancer [23]. Cellular viability was unaffected by 2μM sodium arsenite, 2J/cm^2^ of UV or 50μM nicotinamide, indicating that our interventions did not affect cell death or growth. This is consistent with previous studies demonstrating no reduction in viability with 2μM sodium arsenite [24] or nicotinamide with 4J/cm^2^ of UV [25]. Hence the doses used in this study did not introduce the ambiguity of variable numbers of nonviable cells in the DNA damage and repair assays. The combination of these low-dose UV and arsenic exposures, in both human keratinocytes and in whole human skin, provides a model that can be compared to real life UV and arsenic exposure in at-risk populations.

These doses were however high enough to induce DNA damage. Incubation of keratinocytes with sodium arsenite followed by UV irradiation led to a significant increase in 8oxoG compared to UV alone over the timecourse studied. This is consistent with other studies albeit with different UV sources and doses [26] that arsenite augments UV-induced oxidative damage to DNA. Consistent results were also evident in our ex vivo human skin studies. CPD levels were not increased by arsenite.

Although photolesion formation occurs at or soon after irradiation [27], peak levels of photolesions were detected at 30 minutes for 8oxoG and 45 minutes for CPDs. Delay in detection of photolesions may relate to their accessibility in the context of the DNA repair process [28] and has previously been reported in keratinocytes [26,27]. Repair was subsequently observed over the timecourse chosen with the majority of repair occurring within 75 minutes for 8oxoG, consistent with previous studies [18]. Our current immunohistochemistry findings are also consistent with our previous photolesion repair curves in keratinocytes using Comet assays [18]. The observed levels of 8oxoG in unirradiated cells are also consistent with previous studies [18,29], and likely reflect oxidative DNA damage from background ROS production in living cells subjected to cell processing during experimental procedures.

The exact mechanisms of arsenic’s effects on DNA damage and repair are not clear. Arsenic increases UV-induced skin cancer in mice [9] and has been postulated to inhibit DNA repair [28]. There is evidence that sodium arsenite directly inhibits poly (ADP-ribose) polymerase 1 (PARP-1), an essential enzyme in DNA damage detection [24]. However any detectable arsenic inhibition of DNA repair was limited to repair of 8oxoG as it did not increase the number of CPDs above that of UV alone in these models. Previous studies have shown arsenic dose-responsiveness of DNA repair inhibition for CPDs [14], and it may be that our concentration of arsenic was too low to significantly affect CPD repair. Nicotinamide is a precursor of NAD^+^, the sole substrate of PARP-1 [19]. Sodium arsenite is also thought to increase ROS formation in cells, albeit at higher concentrations than ours [30]. Arsenite was previously reported to diminish cellular antioxidant responses [31], which would also result in formation of more 8oxoG. Recent studies have however suggested that arsenite exposure at concentrations similar to ours may cause some increase in gene expression levels of DNA repair enzymes such as the 8oxoG repair enzyme human 8-oxoguanine DNA N-glycosylase 1 (hOGG1) [10]. Arsenic-induced ROS are also thought to contribute to PARP-1 inhibition and subsequent impairment of DNA repair [32]. Notably, we previously found that nicotinamide does not have intrinsic anti-oxidant effects in irradiated human keratinocytes [19], and it does not act by filtering UV [17], by preventing photolesion formation [18] or by enhancing the expression of hOGG1 [18]. While long-term exposure to arsenite has previously been shown to modulate p53 activation [33], we found no difference in the effects of nicotinamide or short-term arsenite in normal human skin compared to a human keratinocyte line characterised by mutant p53 (HaCaT).

As a precursor of NAD, NAD^+^ and NADP^+^ [34] nicotinamide is central in cellular bioenergetics. Chromatin remodeling and other steps in DNA repair are known to be highly ATP dependent [20]. UV radiation depletes cellular ATP, but nicotinamide at the same concentration as used here prevents this in HaCaT cells [19]. Depletion of NAD also inhibits glycolysis [35], whereas nicotinamide prevents UV-induced glycolytic blockade [19]. Sodium arsenite has also been reported to deplete ATP in HeLa S-3 cells, although at higher doses [36]. Hence nicotinamide is likely to have reduced photolesions in keratinocytes treated with arsenic and UV by increasing ATP availability for DNA repair. This enhancement of DNA repair by nicotinamide has been confirmed by our findings of increased unscheduled DNA synthesis in HaCaT keratinocytes and in normal human melanocytes exposed to UV and nicotinamide compared to UV alone [18,29].

Arsenic is also immunosuppressive [37]. Patients with arsenic-induced Bowens disease have significantly reduced circulating CD4^+^ T cells compared to control patients and show intralesional CD4^+^ apoptosis [38]. UV is also a potent suppressor of cutaneous immunity and this dual hit to the skin in arsenic-affected populations likely contributes further to skin carcinogenesis. Nicotinamide, when given topically or orally, reduces UV immunosuppression [17,39,40]. This immune protective mechanism may also assist in the chemoprevention of arsenical skin cancer.

## Conclusions

These studies add weight to the evidence that arsenic is a co-carcinogen with UV in skin carcinogenesis, likely by adversely influencing DNA repair and or ROS mediated damage. Nicotinamide enhanced DNA repair in these in vitro and ex vivo studies using clinically relevant exposures to UV and to arsenic. Nicotinamide is a widely available, inexpensive vitamin that is a potential chemopreventive agent for arsenic induced skin cancer in at risk populations.

## References

[1] Rahman MM, Chowdhury UK, Mukherjee SC, Mondal BK, Paul K, et al (2001) Chronic arsenic toxicity in Bangladesh and West Bengal, India—a review and commentary. J Toxicol Clin Toxicol 39: 683–700. 11778666 10.1081/clt-100108509

[2] Berg M, Stengel C, Pham TK, Pham HV, Sampson ML, et al (2007) Magnitude of arsenic pollution in the Mekong and Red River deltas—Cambodia and Vietnam. Sci Tot Environ 372: 413–425. 17081593 10.1016/j.scitotenv.2006.09.010

[3] Rodriguez-Lado L, Sun G, Berg M, Zhang Q, Xue H, et al (2013) Groundwater arsenic contamination throughout China. Science 341: 866–868. 10.1126/science.1237484 23970694

[4] Wu MM, Chiou HY, Wang TW, Hsueh YM, Wang IH, et al (2001) Association of blood arsenic levels with increased reactive oxidants and decreased antioxidant capacity in a human population of northeastern Taiwan. Environ Health Perspect 109: 1011–1017. 11675266 10.1289/ehp.011091011 PMC1242077

[5] Centeno JA, Mullick FG, Martinez L, Page NP, Gibb H, et al (2002) Pathology related to chronic arsenic exposure. Environ Health Perspect 110: 883–886. 12426152 10.1289/ehp.02110s5883 PMC1241266

[6] Haque R, Mazumder DN, Samanta S, Ghosh N, Kalman D, et al (2003) Arsenic in drinking water and skin lesions: dose-response data from West Bengal, India. Epidemiol 14: 174–182. 10.1097/01.EDE.0000040361.55051.54 12606883

[7] Fernández MI, López JF, Vivald iB, Coz F (2012) Long-term impact of arsenic in drinking water on bladder cancer health care and mortality rates 20 years after end of exposure. J Urol 187: 856–861. 10.1016/j.juro.2011.10.157 22248521

[8] Halliday GM, Byrne SN, Damian DL (2011) Ultraviolet A radiation: its role in immunosuppression and carcinogenesis. Semin Cutan Med Surg 30: 214–221. 10.1016/j.sder.2011.08.002 22123419

[9] Rossman TG, Uddin AN, Burns FJ, Bosland MC (2001) Arsenite is a cocarcinogen with solar ultraviolet radiation for mouse skin: an animal model for arsenic carcinogenesis. Toxicol Appl Pharmacol 176: 64–71. 11578149 10.1006/taap.2001.9277

[10] Lee C-H, Wu S-B, Hong C-H, Chen G-S, Wei Y-H, et al (2013) Involvement of mtDNA damage elicited by oxidative stress in the arsenical skin cancers. J Invest Dermatol 133: 1890–1900. 10.1038/jid.2013.55 23370535

[11] Ding W, Hudson LG, Liu KJ (2005) Inorganic arsenic compounds cause oxidative damage to DNA and protein by inducing ROS and RNS generation in human keratinocytes. Mol Cell Biochem 279: 105–112. 16283519 10.1007/s11010-005-8227-y

[12] Klein CB, Leszczynska J, Hickey C, Rossman TG (2007) Further evidence against a direct genotoxic mode of action for arsenic-induced cancer. Toxicol Appl Pharmacol 222: 289–297. 17316729 10.1016/j.taap.2006.12.033 PMC1986829

[13] Qin XJ, Liu W, Li YN, Sun X, Hai CX, et al (2012) Poly(ADP-ribose) polymerase-1 inhibition by arsenite promotes the survival of cells with unrepaired DNA lesions induced by UV exposure. Toxicol Sci 127: 120–129. 10.1093/toxsci/kfs099 22387748 PMC3327874

[14] Ding W, Hudson LG, Sun X, Feng C, Liu KJ (2008) As(III) inhibits ultraviolet radiation-induced cyclobutane pyrimidine dimer repair via generation of nitric oxide in human keratinocytes. Free Radic Biol Med 45: 1065–1072. 10.1016/j.freeradbiomed.2008.06.022 18621123 PMC2583127

[15] Surjana D, Halliday GM, Martin AJ, Moloney FJ, Damian DL (2012) Oral nicotinamide reduces actinic keratoses in phase II double-blinded randomized controlled trials. J Invest Dermatol 132: 1497–1500. 10.1038/jid.2011.459 22297641

[16] Moloney FJ, Vestergaard ME, Radojkovic BL, Damian DL (2010) Randomised, double-blinded, placebo controlled study to assess the effect of topical 1% nicotinamide on actinic keratoses. Br J Dermatol 162: 1138–1139. 10.1111/j.1365-2133.2010.09659.x 20199551

[17] Damian DL, Patterson CRS, Stapelberg M, Park J, Barnetson RS, et al (2008) Ultraviolet radiation-induced immunosuppression is greater in men and prevented by topical nicotinamide. J Invest Dermatol 128: 447–454. 17882270 10.1038/sj.jid.5701058

[18] Surjana D, Halliday GM, Damian DL (2013) Nicotinamide enhances repair of ultraviolet radiation-induced DNA damage in human keratinocytes and ex vivo skin. Carcinogen 34: 1144–1149. 10.1093/carcin/bgt017 23349012

[19] Park J, Halliday GM, Surjana D, Damian DL (2010) Nicotinamide prevents ultraviolet radiation-induced cellular energy loss. Photochem Photobiol 86: 942–948. 10.1111/j.1751-1097.2010.00746.x 20492562

[20] Halliday GM, Bock VL, Moloney FJ, Lyons JG (2009) SWI/SNF: A chromatin-remodelling complex with a role in carcinogenesis. Int J Biochem Cell Biol 41: 725–728. 10.1016/j.biocel.2008.04.026 18723114

[21] Damian DL, Halliday GM (2002) Measurement of ultraviolet radiation-induced suppression of recall contact and delayed-type hypersensitivity in humans. Methods 28: 34–45. 12231186 10.1016/s1046-2023(02)00208-6

[22] Ohno M, Oka S, Nakabeppu Y (2009) Quantitative analysis of oxidized guanine, 8-oxoguanine, in mitochondrial DNA by immunofluorescence method. Methods Mol Biol 554: 199–212. 10.1007/978-1-59745-521-3_13 19513676

[23] Ivanov VN, Hei TK (2006) Sodium arsenite accelerates TRAIL-mediated apoptosis in melanoma cells through upregulation of TRAIL-R1/R2 surface levels and downregulation of cFLIP expression. Exp Cell Res 312: 4120–4138. 17070520 10.1016/j.yexcr.2006.09.019 PMC1839882

[24] Ding W, Liu W, Cooper KL, Qin XJ, de Souza Bergo PL, et al (2009) Inhibition of poly(ADP-ribose) polymerase-1 by arsenite interferes with repair of oxidative DNA damage. J Biol Chem 284: 6809–6817. 10.1074/jbc.M805566200 19056730 PMC2652344

[25] Javeri A, Huang XX, Bernerd F, Mason RS, Halliday GM (2008) Human 8-oxoguanine-DNA glycosylase 1 protein and gene are expressed more abundantly in the superficial than basal layer of human epidermis. DNA Repair (Amst) 7: 1542–1550. 10.1016/j.dnarep.2008.05.011 18585103

[26] Snopov SA, de Gruijl FR, Roza L, van der Leun JC (2004) Immunochemical study of DNA modifications in the nuclei of UV-damaged lymphocytes. Photochem Photobiol Sci 3: 85–90. 14743284 10.1039/b305135h

[27] Gupta R, Dixon KM, Deo SS, Holliday CJ, Slater M, et al (2007) Photoprotection by 1,25 dihydroxyvitamin D3 is associated with an increase in p53 and a decrease in nitric oxide products. J Invest Dermatol 127: 707–715. 17170736 10.1038/sj.jid.5700597

[28] Wu F, Burns FJ, Zhang R, Uddin AN, Rossman TG (2005) Arsenite-induced alterations of DNA photodamage repair and apoptosis after solar-simulation UVR in mouse keratinocytes in vitro. Environ Health Perspect 113: 983–986. 16079067 10.1289/ehp.7846 PMC1280337

[29] Thompson BC, Surjana D, Halliday GM, Damian DL (2014) Nicotinamide enhances repair of ultraviolet radiation-induced DNA damage in primary melanocytes. Exp Dermatol: 10.1111/exd.12620 24798949

[30] Shi H, Hudson LG, Ding W, Wang S, Cooper KL, et al (2004) Arsenite causes DNA damage in keratinocytes via generation of hydroxyl radicals. Chem Res Toxicol 17: 871–878. 15257611 10.1021/tx049939e

[31] Sun Y, Kojima C, Chignel C, Mason RS, Waalkes M (2011) Arsenic transformation predisposes human skin keratinocytes to UV-induced DNA damage yet enhances their survival apparently by diminishing oxidant response. Toxicol Appl Pharmacol 255: 242–250. 10.1016/j.taap.2011.07.006 21820459 PMC3169845

[32] Wang F, Zhou XF, Liu W, Sun X, Chen C, et al (2013) Arsenite-induced ROS/RNS generation causes zinc loss and inhibits the activity of poly(ADP-ribose) polymerase-1. Free Rad Biol Med 61: 249–256. 10.1016/j.freeradbiomed.2013.04.019 23602911 PMC3766412

[33] Komissarova EV, Rossman TG (2010) Arsenite induced poly(ADP-ribosyl)ation of tumor suppressor P53 in human skin keratinocytes as a possible mechanism for carcinogenesis associated with arsenic exposure. Toxicol Appl Pharmacol 243: 399–404. 10.1016/j.taap.2009.12.014 20036271 PMC2830301

[34] Belenky P, Bogan KL, Brenner C (2007) NAD(+) metabolism in health and disease. Trends Biochem Sci 32: 12–19. 17161604 10.1016/j.tibs.2006.11.006

[35] Jacobson EL, Giacomoni PU, Roberts MJ, Wondrak GT, Jacobson MK (2001) Optimizing the energy status of skin cells during solar radiation. J Photochem Photobiol B 63: 141–147. 11684461 10.1016/s1011-1344(01)00211-1

[36] Yih LH, Huang HM, Jan KY, Lee TC (1991) Sodium arsenite induces ATP depletion and mitochondrial damage in HeLa cells. Cell Biol Int Rep 15: 253–264. 2032293 10.1016/0309-1651(91)90157-e

[37] Lee CH, Liao WT, Yu HS (2011) Aberrant immune responses in arsenical skin cancers. Kaohsiumg J Med Sci 27: 396–401. 10.1016/j.kjms.2011.05.007 21914527 PMC11916620

[38] Liao WT, Yu CL, Lan CC, Lee CH, Chang CH, et al (2009) Differential effects of arsenic on cutaneous and systemic immunity: focusing on CD4+ cell apoptosis in patients with arsenic-induced Bowen’s disease. Carcinogen 30: 1064–1072. 10.1093/carcin/bgp095 19376847

[39] Yiasemides E, Sivapirabu G, Halliday GM, Park J, Damian DL (2009) Oral nicotinamide protects against ultraviolet radiation-induced immunosuppression in humans. Carcinogen 30: 101–105. 10.1093/carcin/bgn248 19028705

[40] Sivapirabu G, Yiasemides E, Halliday GM, Park J, Damian DL (2009) Topical nicotinamide modulates cellular energy metabolism and provides broad-spectrum protection against ultraviolet radiation-induced immunosuppression in humans Br J Dermatol 161: 1357–1364. 19804594 10.1111/j.1365-2133.2009.09244.x

